# Assessing organizational readiness for the Clean Cuts and Sharp Minds Collective: a barbershop health promotion network

**DOI:** 10.1186/s43058-024-00584-x

**Published:** 2024-04-16

**Authors:** Guillermo M. Wippold, Zion R. Crichlow, Kaylyn A. Garcia, Ariel Domlyn, Shane Sanchez, Lucina Frank, Thrisha Mote, Sarah Grace Frary, Terry Woods

**Affiliations:** 1 Department of Psychology, University of South Carolina, 1512 Pendleton Avenue, Barnwell College, Mailbox 38, Columbia, SC 29208 USA; 2 Center for Clinical Management Research, VA Ann Arbor Healthcare System, Ann Arbor, MI USA; 3 Main Attraction Barbershop, Sumter, SC USA; 4 Healthy Mind, Body, and Family Foundation, Sumter, SC USA

**Keywords:** Black men, African American men, Barbershops, Readiness, Partnership, Health equity, Health promotion

## Abstract

**Background:**

Black men have among the lowest life expectancy in the United States. Alarmingly, these men are underrepresented in health promotion efforts. There are well-documented barriers to recruiting and retaining Black men in health promotion efforts, such as exclusionary research practices – many researchers may be hesitant to reach Black men in culturally unique spaces, such as barbershops. Despite these practices, qualitative research among Black men unanimously find that Black men are interested in health promotion efforts. The Clean Cuts and Sharp Minds Collective (CCSMC) was designed to bridge this gap. The objectives of the CCSMC are to train barbers to be lay advocates for their clients, train barbers to be research partners, and serve as a nexus between barbers interested in health promotion at their shops and researchers interested in implementing such efforts. The present study sought to assess the organizational readiness of barbershops in South Carolina (SC) to participate in the CCSMC.

**Methods:**

Barbers in SC were invited to complete a modified version of the Readiness Thinking Tool to assess organizational readiness to participate in the CCSMC.

**Results:**

Thirty-six (36; mean age = 41.12; 94.4% identified as Black; 91.7% identified as male) barbers completed the organizational readiness assessment. Results indicated that there was a high level of motivation, innovation-specific capacity, and general capacity within barbershops to participate in the CCSMC. Additionally, many barbers indicated that there would be widespread support to join the CCSMC.

**Conclusions:**

The results from the present study highlight exciting opportunities and future directions for barbershop-academic partnerships. Such partnerships have the potential to promote health equity among, and in partnership with, Black men.

**Supplementary Information:**

The online version contains supplementary material available at 10.1186/s43058-024-00584-x.

Contributions to the literature

- Builds on existing community-based health promotion models by demonstrating the potential of utilizing culturally relevant community settings such as barbershops to promote Black men’s health.
- Underscores the importance of capacity building for sustainable barbershop-based health promotion efforts.
- Seeks to address gaps in health equity and access for Black men in the United States.
- Identifies concrete strategies for overcoming barriers to implementation of barbershop-based health promotion efforts, which have been hindered by exclusionary practices.
- Describes an innovative approach to foster future barbershop-academic partnerships to improve Black men’s health.

## Background

### Health among Black men

Black men have among the lowest life expectancy of any racial/ethnic-gender group in the United States (U.S.) [1]. Black men have a life expectancy of 67.8 years, which is 13.3 years less than Asian men, 7 years less than White men, and 6.8 years less than Hispanic men in the U.S. [1]. Leaders in health promotion among Black men have identified many causes of adverse health and premature mortality among Black men [2]. These causes include public health concerns such as high rates of unfavorable physical health conditions (e.g., cardiovascular disease, diabetes, some forms of cancer), mental health conditions (e.g., stress, depression [3]), and social determinants of health (e.g., gender socialization), as well as discrimination, access to health care, employment, and income [2]. Many of these causes have also been linked to lower health-related quality of life among Black adults [4–6]. Therefore, efforts are urgently needed to promote health among, and in partnership with, Black men.

### Participation of Black men in health promotion activities

It has been 30 years since the 1993 Revitalization Act of the National Institutes of Health (NIH) – a mandate that all clinical trials should include a proportional representation of racial-ethnic minoritized individuals [7]. Despite this national mandate, a recent report (2022) from the National Academies of Science, Engineering, and Medicine (NASEM) concluded that demographic trends of federally funded clinical research have not changed [8, 9]. This is supported by evidence that since this mandate, fewer than 2% of those enrolled in clinical cancer trials are individuals from racial-ethnic minoritized backgrounds [10] and less than 5% of NIH-funded research on respiratory health report the inclusion of individuals from marginalized backgrounds [11]. Similarly low rates can be found in cardiovascular and diabetes research [12, 13]. Although the low rate of inclusion of individuals from racial-ethnic minoritized backgrounds is alarming, it has been suggested that the inclusion of Black men in health promotion research is much lower [14].

Due to this low rate of participation, the health promotion literature has often dubbed Black men a “hard-to-reach population” – a designation that places undue blame on Black men as opposed to the methods being used to recruit, retain, and produce meaningful health-related change. It should be recognized that there is rational mistrust of health promotion researchers among Black Americans because of studies such as the Tuskegee Syphilis Study and that this mistrust contributes to low participation rates [15, 16]. With that being the case, researchers and staff must also recognize their own role in contributing to the low rate of participation in health promotion efforts among Black Americans. There is a growing body of literature examining barriers to participation in health promotion research by individuals from racial-ethnic minoritized backgrounds due to exclusionary recruitment practices by researchers and staff [9, 17]. It has been found that: (1) researchers find recruitment interactions with potential racial-ethnic minoritized participants as challenging, (2) potential racial-ethnic minoritized participants are often not perceived as ideal study candidates, and (3) that a combination of structural barriers and negative perceptions of racial-ethnic minoritized participants have led researchers to withhold health promotion opportunities from minoritized individuals [17].

The disparities in health outcomes and exclusionary practices serving as barriers to participation in health promotion efforts among individuals from racial-ethnic minoritized backgrounds are dire and have alarming implications. Not only is progress toward health equity hindered, but it has been estimated that eliminating racial/ethnic health disparities would have reduced total medical costs during a three-year period by more than $1.2 trillion [18, 19]. Additionally, according to the NASEM, the consequences of this underrepresentation in health promotion efforts include: (1) compromised generalizability of clinical research findings; (2) hundreds of billions of dollars in health care costs; (3) hindered innovation and new discoveries; (4) low accrual that causes many trials to fail; (5) lack of access to effective interventions; (6) undermined trust of health promotion efforts; and (7) compounded health disparities [8]. Thus, strategies to reverse the trend of underrepresented groups in research are urgently needed [9].

### Barbershop-based health promotion efforts

Qualitative research conducted among Black men dispels the “hard-to-reach population” narrative. This research resoundingly indicates that Black men value their health [20–23] – a necessary precursor to health promotion engagement [24]. This valuing of health among Black men as a necessary precursor to engagement is underscored by the success of interventions implemented in culturally important community settings [25, 26], such as barbershops [27]. Barbershops are a place of cultural significance for many Black men and have long been safe spaces for Black men to congregate and discuss important topics [28], such as health and health promotion. Recently, health promotion efforts conducted by researchers and medical professionals have leveraged the historical importance of barbershops and have successfully improved blood pressure [29, 30], prostate cancer awareness [31], and rates of sexual risk behaviors [32]. A comprehensive review of barbershop-based health promotion programs for Black men applied the RE-AIM Framework to these interventions and found that these efforts were likely to result in satisfactory recruitment and retention rates and produce health-related changes when they (a) prioritized community engagement and (b) intentionally aligned with gender- and race-based lived experiences [27].

### Clean Cuts and Sharp Minds Collective

The Clean Cuts and Sharp Minds Collective (CCSMC) was developed to increase barbershop-academic partnered health promotion efforts. The CCSMC is a community-identified and supported solution to advance Black men’s health by developing a network of trained barbers to promote clients’ health and engagement in research. The CCSMC is an innovative initiative leveraging barbershops as health promotion hubs for Black men. The CCSMC taps into the trusted barber-client relationship, utilizing barbers as health advocates to address health equity issues. By aligning health strategies with Black men's values and preferences, the CCSMC aims to boost engagement in health-related activities and participation in research, fostering a community-driven approach to improving health outcomes and reducing disparities among Black men.

The CCSMC recognizes that barbershops have been sources of health promotion and discussions for Black men for generations. However, these discussions are infrequently guided by informed health advocates, missing opportunities for impactful health education and intervention. Additionally, many barbers are hesitant to engage with health promotion researchers. The CCSMC initiative seeks to bridge this gap by (1) training barbers to be lay advocates for their clients, (2) training barbers to be research partners, and (3) serving as a nexus between barbers interested in health promotion at their shops and researchers interested in implementing such efforts.

### Objective and organizational readiness

Ravenell and colleagues have indicated that there is an evidence gap in understanding whether community-based settings, such as barbershops, have the capacity to coordinate the complex set of changes required for adopting and sustaining health promotion interventions and programs [33], such as those of the CCSMC. The objective of the present manuscript is to report on the organizational readiness of barbershops to participate in the CCSMC. Although there are various definitions of organizational readiness [34, 35], one common conceptualization refers to the motivation, general organizational capacity, and innovation-specific capacity of an organization to implement a change [35]. When organizational readiness is high, organization members are more likely to institute change, dedicate more time, exhibit greater persistence, and display more cooperative behavior, leading to effective implementation of the proposed innovation [36]. Findings indicate that failure to establish organizational readiness accounts for half of all unsuccessful organizational change efforts [34, 37]. Organizational readiness is both an indicator of preparedness for conducting health promotion efforts and a malleable state that can indicate needs for capacity building [38–40]. Thus, the purpose of the present study was to conduct a survey-based organizational readiness assessment among barbershops that serve predominantly Black men in the state of South Carolina to implement culturally tailored health promotion interventions and programs in the barbershop. Information gathered will indicate either existing capacity for successful health promotion partnerships or areas to prioritize for bolstering motivation and capacity to enhance success.

## Methods

### Participants

To be eligible for participation, participants must 1) be 18 years of age or older, 2) be a barber in the state of South Carolina, and 3) work at a barbershop that serves predominantly Black male clientele.

### Measures

A modified version of the Readiness Thinking Tool (RTT) was used [35]. See Supplementary File 1. The RTT is designed to assess an organization’s readiness to implement a program, policy, practice, or process. Respondents were asked to indicate whether they disagree, partially agree, strongly agree, or are unsure about 18 items assessing motivation (degree to which an organization wants the innovation to happen), innovation-specific capacity (what is needed to make this particular innovation happen), and general capacity (the organization’s overall functioning). Items were modified to specifically assess readiness to promote health in the respondent’s barbershop. A sample item is “It seems easy to promote health at our barbershop.” The Cronbach’s alpha of this measure is .85 indicating strong reliability for the current sample.

### Procedure

Following Institutional Review Board approval, a list of licensed and insured barbershops was obtained. The research team reviewed the list to identify barbershops and then researched selected barbershops to determine if they predominately serve Black men – as evidenced by images of predominantly Black male clientele on business websites and/or social media profiles. Convenience and snowball sampling methodologies were also used to recruit participants. Specifically, the research team recruited from barbershops in which pre-established research partnerships existed. These barbershops also connected the research team to other barbershops. Additionally, flyers with a QR code to the survey were printed and distributed to local beauty supply stores and posted around a university campus. Flyers were also posted on social media (i.e., Instagram) and sent via direct message to barbers from the research team’s Instagram page. Identified barbershops (or individual barbers) were then contacted either by phone, email, in person, and/or via social media direct message or video call. Barbers who were recruited in person or via phone or social media were asked if they would be willing to complete a short online survey (i.e., the modified RTT) about their perspective on promoting health in barbershops. The modified RTT was administered via Qualtrics and consenting participants were given the option to complete the survey in person, over the phone with a member of the research team, or be sent a link to the survey via text or email. At the start of the survey, participants were provided a brief description of the study and were asked to confirm they work in a barbershop that predominantly serves Black male clientele. Consenting participants proceeded with items from the modified RTT and ended the survey with demographic questions. The median number of minutes it took to complete the survey was seven and participants were not compensated for their participation.

## Results

### Participants

A total of 40 barbers participated in the survey, in which 36 met inclusion criteria and are included in the following analyses. Most barbers identified as Black (94.4%), followed by White (5.6%), and another racial identity (unspecified) (2.8%). Most barbers identified as male (91.7%), followed by female (8.3%). The average age was 41.42 (*SD* = 11.4) years old and reported an average of 17.53 (*SD* = 13.56) years of experience as a barber. The average number of clients seen per week by each barber was 57.41 (*SD* = 40.78). The number of barbers at each barbershop was 4.17 (*SD* = 2.14). Participants represented 25 unique barbershops across the state of South Carolina (i.e., in some instances, multiple barbers from the same barbershop completed the survey).

### Innovation information

Barbers were presented with a brief description of a plan to establish a network of barbershops interested in promoting men’s health (i.e., the CCSMC). They were then asked a series of question about the innovation. See Table 1 and below.

**Table 1:** Responses to questions about the Cleans Cuts and Sharp Minds Collective

Question	% yes
“In general, do you think the Clean Cuts and Sharp Minds Collective is a good idea?”	100
“Do you think barbers would also be interested in learning how to promote health among their clients in partnership with other groups/organizations such as community-based health promotion researchers?”	100
Would you regularly attend the Clean Cuts and Sharp Minds Collective meetings?	86.1

The results indicate that 100% of the barbers indicated that the CCSMC was a good idea and that 100% of barbers would be interested in learning how to promote health among their clients in partnership with other organizations, such as community-based health promotion researchers. Finally, the vast majority (i.e., 86.1%) of barbers indicated that they would regularly attend CCSMC meetings. They were then asked “The Clean Cuts and Sharp Minds Collective will meet virtually over Zoom to discuss ways to promote health in the barbershop. How often do you think the Clean Cuts and Sharp Minds Collective should meet?” 30.6% barbers recommended every two weeks and 69.4% recommended every month. Finally, barbers were asked “If we asked 10 barbershops to participate in the Clean Cuts and Sharp Minds Collective, how many of the 10 do you think would actually participate in the Clean Cuts and Sharp Minds Collective?” The mean response was 7 (*SD* = 2.05).

### Organizational readiness to implement innovation

See Supplementary File 1 for the list of questions used to assess each indicator of organizational readiness. Regarding the Motivation domain of the RTT, the majority of participants (i.e., > 86.1%) partially agreed or strongly agreed that: (1) promoting health at the barbershop seems better than focusing on the cut or talking about other things (i.e., Relative Advantage); (2) promoting health at the barbershop fits with the barbershop’s mission and clients’ needs (i.e., Compatibility); (3) it seems easy to promote health at the barbershop (i.e., Simplicity); (4) it is possible to try to promote health at the barbershop on a small scale to see how it goes (i.e., Ability to Pilot); (5) over time, they will be able to see how promoting health at the barbershop helps clients be healthier (i.e., Observability); and (6) promoting health at the barbershop is a priority (i.e., Priority). See Table 2 for the results of the Motivation domain.

**Table 2:** Motivation domain

% Agreement degree to which an organization wants the innovation to happen.				
	Disagree	Partially Agree	Strongly Agree	Unsure
Relative Advantage	8.3	36.1	50	5.6
Compatibility	2.8	16.7	80.6	-
Simplicity	11.1	22.2	66.7	-
Ability to Pilot	-	13.9	86.1	-
Observability	-	11.1	88.9	2.5
Priority	2.8	27.8	66.7	2.8

Regarding the Innovation-Specific Capacity domain, the majority of participants (i.e., > 88.9%) partially agreed or strongly agreed that: (1) the barbershop has the ability to promote health among clients (i.e., Innovation-Specific Knowledge & Skills); (2) there is staff at the barbershop that could support barbers’ learning to promote health among their clients (i.e., Champion); (3) the barbershop has the time and interest to learn how to promote health among their clients (i.e., Supportive Climate); (4) the barbershop has good relationships with other business or nearby organizations that could help them promote health among their clients (i.e., Inter-Organizational Relationships); and (5) the staff could support each other in promoting health among their clients (i.e., Intra-Organizational Relationships). See Table 3 for the results of the Innovation-specific Capacity domain.

**Table 3:** Innovation-specific Capacity domain

% Agreement what is needed to make this particular innovation happen.				
	Disagree	Partially Agree	Strongly Agree	Unsure
Innovation-specific Knowledge & Skills	-	13.9	86.1	-
Champion	8.3	22.2	66.7	2.8
Supportive Climate	-	41.7	55.6	2.8
Inter-organizational Relationships	5.6	22.2	66.7	5.6
Intra-organizational Relationships	-	13.9	86.1	-

Regarding the General Capacity domain, the majority of participants (i.e., > 86.1%) partially agreed or strongly agreed that: (1) the barbershop has a shared vision, mission, and way of doing things (i.e., Culture); (2) staff at the barbershop feel good about working at the barbershop (i.e., Climate); (3) the barbershop is open to improvement (i.e., Innovativeness); (4) the barbershop has resources that can be utilized (i.e., Resource Utilization); (5) the barbershop has good leaders (i.e., Champion); (6) the barbershop has good communication and generally functions well (i.e., Internal Operations); and (7) the barbershop has enough of the right kind of staff to get things done (i.e., Staff Capacities). See Table 4 for results from the General Capacity domain.

**Table 4:** General Capacity domain

% Agreement the organization’s overall functioning.				
	Disagree	Partially Agree	Strongly Agree	Unsure
Culture	5.6	22.2	72.2	-
Climate	-	5.6	94.4	-
Innovativeness	-	-	100	-
Resource Utilization	2.8	25.0	61.1	11.1
Champion	-	5.6	94.4	-
Internal Operations	-	5.6	94.4	-
Staff Capacities	-	5.6	94.4	-

## Discussion

Black men experience high rates of adverse health conditions that contribute to a low life expectancy [1]. Alarmingly, health promotion efforts among Black men have been met with difficulties in recruitment, retention, and the production of meaningful health-related change. These difficulties have led Black men to be referred to as a “hard-to-reach” population – a designation that places undue blame on Black men as opposed to the exclusionary practices by researchers. Qualitative research resoundingly indicates that Black men care about and value their health [20–22]. This has been evidenced by the widespread success of barbershop-based efforts to promote health among Black men [27, 41, 42]. The present study sought to contribute to this body of research by addressing a critical gap identified by Ravenell and colleagues [33] – whether barbershops have the capacity to implement health promotion programming in partnership with researchers. Specifically, the present study sought to assess whether barbershops have the organizational readiness to take part in the Clean Cuts and Sharp Minds Collective (CCSMC) – a network that seeks to bridge the gap between Black men’s desire to engage in health promotion efforts and the lack of barbershop-academic partnerships.

The findings from the present study show overwhelming support and optimism for the CCSMC among barbers. The response to the CCSMC’s concept was unanimously positive – 100% of barbers agreed that it was a good idea and that their barber-peers would be interested in participating. Additionally, barbers estimated that 70% of other barbers approached to participate in the CCSMC would likely agree to do so. This high level of anticipated engagement and uptake indicates a strong interest in the CCSMC and a positive outlook on its practicality and potential success [43]. This positive outlook highlights the exciting potential of community-centered implementation models to promote health among Black men. Rigorous reviews have found that programming implemented in community venues (e.g., barbershops, churches) play an important role in health promotion among Black men [27, 44]. Such efforts (e.g., CCSMC) are often tailored to surface-level and deep-level cultural considerations [45]. This tailoring is known to promote engagement and uptake [44].

The results from the present study also indicate that most barbers agree that their barbershops have sufficient organizational motivation to participate in the CCSMC. This suggests that barbers see clear benefits in the CCSMC, believe that the CCSMC aligns well with their values, feel that the CCSMC is manageable and can be tested on a small scale before large scale implementation, and that outcomes would be noticeable. These results provide evidence that adapting and tailoring a health promotion program (e.g., CCSMC) for Black men by aligning the program to the preferences, perspectives, and values of Black men may contribute to high levels of motivation to participate. Additionally, the results indicate that barbers feel confident in their ability and knowledge to implement CCSMC initiatives and that barbershops have cohesive environments that would facilitate the CCSMC initiatives. Combined with the aforementioned high level of motivation, this high level of capacity indicates that health promotion programming can be successfully implemented in the barbershop. Interestingly, there was moderate support for having a champion and supportive climate. These areas may require additional capacity building and could strengthen the implementation of the CCSMC and other similar health promotion efforts. Finally, results show that barbershops have a positive and supportive environment, which is critical to capacity and sustainability of these efforts. Finally, the results indicate that barbershops are also well-equipped to adapt and efficiently use their resources for health promotion initiatives such as the CCSMC.

### Future directions

In addition to addressing a significant gap in the literature, the results from the present study provide insight into exciting future directions for barbershop-based health promotion efforts among, and in partnership, with Black men. Although there is documented widespread support for the effectiveness of barbershop-based health promotion efforts [27, 42, 46], and now indicators of strong organizational readiness to implement such efforts, there remains a need for (1) implementation studies, (2) policy and funding models, and (3) strategies to foster greater organizational readiness. Addressing such needs will contribute to successful efforts and health equity – a goal of implementation science [47]. Barbershop-based health promotion efforts remain a “black box” [27] – that is, there has been a significant focus on health outcomes (e.g., hypertension, cancer screening rates) of these efforts as opposed to the processes (e.g., partnership development, capacity building, sustainability) used to achieve those outcomes [48], though findings from one review indicate that barbershop-based efforts emphasizing community engagement and race/gender-based tailoring are successful in recruiting, retaining, and producing health-related changes [27]. Implementation studies are needed to identify best practices and potential challenges to these efforts, including strategies to maximize community engagement and race/gender-based tailoring (e.g., attending to surface-level and deep-level cultural considerations [45]). Additionally, there is a need for policy and funding models to support sustainable health promotion efforts in barbershops. It is evident that Black men experience high rates of adverse health concerns and that barbershop-based health promotion efforts are effective for Black men; thus, policy and funding cycles that prioritize engagement and ensure that these efforts are sustained and scaled are needed. Finally, there is a continued need to understand specific strategies that can foster organizational readiness among organizations [49], such as barbershops. Likewise, given the growing [50]interest in exclusionary practices by researchers, there is a need to understand researchers’ readiness to conduct barbershop-based health promotion programs. Similar efforts have been made in intermediary support organizations, where technical assistance providers’ readiness for supporting health efforts was assessed and improved [50]. A similar effort could be made for the readiness of researchers to commit to deep partnerships with barbershops. A thorough assessment could be followed by readiness training that prepares researchers to engage with culturally significant settings, such as the barbershop, in an effective and respectful manner.

### Limitations and strengths

The results and suggested future directions should be viewed in conjunction with the limitations and strengths of the present study. There are two noteworthy limitations: (1) geographic limits to generalizability and (2) potential response bias. It should be noted that the present study occurred in South Carolina and all respondents were from barbershops in South Carolina that serve predominantly Black men. This may limit the generalizability of the findings given that other states may have varying cultural and social contexts that may impact organizational readiness. With that being the case, there have been calls for intersectional understandings of men’s health [51] and the Deep South (where South Carolina is located) is an area marked by disproportionately high rates of adverse health [52]. Such health promotion efforts are urgently needed. Additionally, the present study may have also been impacted by response bias, or the reporting of socially desirable responses. It may be the case that only barbershops inclined to participate in the CCSMC completed the organizational readiness assessment. With that being the case, all participants were notified about the confidentiality of the survey. The strengths of the present study include: (1) targeted community engagement and (2) barber involvement. Focusing exclusively on barbershops allows for precision public health and prevention efforts that can be developed and implemented in partnership with barbershops. Precision prevention posits that tailoring health interventions will be more effective than non-tailored approaches [53, 54]. Additionally, it should be noted that although the present study was initiated by an academic team, it was also guided by the input of a Master Barber, who is an author of this paper and co-founder of the CCSMC. This allowed for the survey to be tailored to both surface-level and deep-level cultural considerations of Black men and barbers [45].

## Conclusions

Black men experience high rates of adverse health and have low rates of representation in health promotion efforts. Although many successful health promotion efforts for Black men have occurred in barbershops, there have been concerns that barbershops may not have the organizational capacity to engage in such efforts. Organizational readiness is often not assessed before implementing a health promotion effort, and a lack of organizational readiness can result in health promotion programming that is not sustainable. Our study found that barbershops serving predominately Black male clientele have the motivation and capacity (i.e., organizational readiness) to implement health promotion programming such as the CCSMC. This is noteworthy because organizational readiness is positively associated with retention and group cohesion and negatively associated with intent to attrite [55]. Thus, such efforts have the high potential to promote health equity among, and in partnership, with Black men.

### Supplementary Information

**Supplementary Material 1.** 

## Data Availability

The datasets used during the current study are available from the corresponding author on reasonable request.

## Abbreviations

CCSMC: Clean Cuts and Sharp Minds Collective
SC: South Carolina
NIH: National Institutes of Health
NASEM: National Academies of Science, Engineering, and Medicine
SD: Standard Deviation
RTT: Readiness Thinking Tool

## References

[1] Arias E, Xu J (2022). United States Life Tables, 2020. Natl Vital Stat Rep.

[2] Gilbert KL, Ray R, Siddiqi A, et al. Visible and Invisible Trends in Black Men's Health: Pitfalls and Promises for Addressing Racial, Ethnic, and Gender Inequities in Health. Ann Rev Public Health. 2016;37:295–311. 10.1146/annurev-publhealth-032315-021556 PMC6531286 26989830

[3] King KM, Key-Hagan M, Desai A (2022). Stress correlates related to depressive symptoms among young black men in Southern California. Am J Men's Health.

[4] Wippold GM, Tucker CM, Roncoroni J, Henry MA. Impact of stress and loneliness on health-related quality of life among low income senior African Americans. J Racial Ethn Health Disparities. 2020;8(4):1089–97. 10.1007/s40615-020-00865-w 32940896

[5] Wippold GM, Garcia KA, Frary SG. The role of sense of community in improving the health-related quality of life among Black Americans. J Commun Psychol. 2022;51(1):251–69. 10.1002/jcop.22901 PMC9742166 35700438

[6] Wippold GM, Frary SG. Predictors of health-related quality of life among African American men. J Racial Ethn Health Disparities. 2021;9(6):2131–8. 10.1007/s40615-021-01151-z PMC8926934 34533780

[7] United States C. National Institutes of Health Revitalization Act of 1993 : conference report (to accompany S. 1). [Washington, D.C.?] : [U.S. G.P.O.], [1993]; 1993.

[8] National Academies of Sciences E, Medicine. Improving Representation in Clinical Trials and Research: Building Research Equity for Women and Underrepresented Groups. Washington, DC: The National Academies Press; 2022. 36137057

[9] Passmore SR, Kisicki A, Gilmore-Bykovskyi A, Green-Harris G, Edwards DF (2022). "There's not much we can do…" researcher-level barriers to the inclusion of underrepresented participants in translational research. J Clin Transl Sci.

[10] Chen MS, Lara PN, Dang JHT, Paterniti DA, Kelly K (2014). Twenty years post- NIH revitalization act: enhancing minority participation in clinical trials ( EMPaCT): laying the groundwork for improving minority clinical trial accrual. Cancer.

[11] Burchard EG, Oh SS, Foreman MG, Celedón JC (2015). Moving toward true inclusion of racial/ethnic minorities in federally funded studies. A key step for achieving respiratory health equality in the United States. Am J Respir Crit Care Med.

[12] Sardar MR, Badri M, Prince CT, Seltzer J, Kowey PR (2014). Underrepresentation of women, elderly patients, and racial minorities in the randomized trials used for cardiovascular guidelines. JAMA Intern Med.

[13] Chow EA, Foster H, Gonzalez V, McIver L (2012). The disparate impact of diabetes on racial/ethnic minority populations. Clin Diabetes.

[14] Graham LF, Scott L, Lopeyok E, Douglas H, Gubrium A, Buchanan D (2018). Outreach strategies to recruit low-income African American men to participate in health promotion programs and research: lessons from the Men of Color Health Awareness (MOCHA) project. Am J Men's Health.

[15] Alsan M, Wanamaker M (2018). Tuskegee and the health of black men. Q J Econ.

[16] Tucker CM, Wippold GM, Guastello AD, et al. Predictors of Cancer Screening Among Culturally Diverse Men. Am J Mens Health. 2018;12(4):837–43. 10.1177/1557988316644398 PMC6131431 27118456

[17] Niranjan SJ, Martin MY, Fouad MN, et al. Bias and stereotyping among research and clinical professionals: Perspectives on minority recruitment for oncology clinical trials. J Clin Oncol. 2019;37(27_suppl):152-152. 10.1002/cncr.32755 32147815

[18] Oh SS, Galanter J, Thakur N (2015). Diversity in Clinical and Biomedical Research: A Promise Yet to Be Fulfilled. Plos Med.

[19] LaVeist TA, Gaskin D, Richard P (2011). Estimating the economic burden of racial health inequalities in the United States. Int J Health Serv.

[20] Heeren GA, Jemmott JB (2011). Health promotion: results of focus groups with African-American men. J Mens Health.

[21] Robinson D, Valdez L, Scott L, Buchanan D (2021). The role of work in gender identity, stress and health in low-income, middle-aged African-American men. Health Promot Int.

[22] Ravenell JE, Johnson WE, Whitaker EE (2006). African-American men's perceptions of health: a focus group study. J Natl Med Assoc..

[23] Tucker CM, Williams JL, Wippold GM, et al. Views of diverse primary care patients on the roles of healthcare providers and staff and the influence of other variables in their weight management. Clin Obes. 2018;8(1):11–20. 10.1111/cob.12225 PMC5760475 29052345

[24] Pender NJ, Murdaugh CL, Parsons MA. Health promotion in nursing practice. 2006.

[25] Tucker CM, Wippold GM, Roncoroni J (2022). Impact of the Health-Smart Holistic Health Program: A CBPR Approach to Improve Health and Prevent Adverse Outcomes for Black Older Adults. J Prev Health Promot.

[26] Tucker CM, Wippold GM, Williams JL, Arthur TM, Desmond FF, Robinson KC (2017). A CBPR study to test the impact of a church-based health empowerment program on health behaviors and health outcomes of black adult churchgoers. J Racial Ethn Health Disparities.

[27] Wippold GM, Frary SG, Garcia KA, Wilson DK (2023). Implementing barbershop-based health-promotion interventions for Black men: a systematic scoping review. Health Psychol.

[28] Mills QT (2013). Cutting along the color line : Black barbers and barber shops in America.

[29] Hess PL, Reingold JS, Jones J (2007). Barbershops as hypertension detection, referral, and follow-up centers for black men. Hypertension.

[30] Victor RG, Ravenell JE, Freeman A, et al. Effectiveness of a barber-based intervention for improving hypertension control in black men - The BARBER-1 study: a cluster randomized trial. Arch Internal Med. 2011;171(4):342–50. 10.1001/archinternmed.2010.390 PMC3365537 20975012

[31] Luque JS, Rivers BM, Gwede CK, Kambon M, Green BL, Meade CD (2011). Barbershop communications on prostate cancer screening using barber health advisers. Am J Men's Health.

[32] Wilson TE, Gousse Y, Joseph MA (2019). HIV prevention for black heterosexual men: the barbershop talk with brothers cluster randomized trial. Am J Public Health.

[33] Ravenell J, Green T, Arabadjian M, Schoenthaler A, Ogedegbe O (2023). Barbershop-facilitated community-to-clinic linkage implementation program: rationale and protocol for a novel program to prevent hypertension among black men. Am J Hypertens.

[34] Weiner BJ (2009). A theory of organizational readiness for change. Implement Sci.

[35] Scaccia JP, Cook BS, Lamont A (2015). A practical implementation science heuristic for organizational readiness: R = MC(2). J Commun Psychol.

[36] Weiner BJ, Lewis MA, Linnan LA (2009). Using organization theory to understand the determinants of effective implementation of worksite health promotion programs. Health Educ Res.

[37] Kotter JP. Leading change: Why transformation efforts fail. 2007.

[38] Livet M, Blanchard C, Richard C (2022). Readiness as a precursor of early implementation outcomes: an exploratory study in specialty clinics. Implement Sci Commun.

[39] Domlyn AM, Scott V, Livet M (2021). R = MC(2) readiness building process: a practical approach to support implementation in local, state, and national settings. J Community Psychol.

[40] Macauda MM, Arent MA, Sakhuja M (2022). Elements for successful implementation of a clinic-based health literacy intervention. Front Public Health.

[41] Palmer KNB, Rivers PS, Melton FL (2021). Health promotion interventions for African Americans delivered in U.S. barbershops and hair salons- a systematic review. BMC Public Health.

[42] Luque J, Ross L, Gwede C (2014). Qualitative systematic review of barber-administered health education, promotion, screening and outreach programs in African-American communities. J Commun Health.

[43] O’Mara-Eves A, Brunton G, Oliver S, Kavanagh J, Jamal F, Thomas J (2015). The effectiveness of community engagement in public health interventions for disadvantaged groups: a meta-analysis. BMC Public Health.

[44] Wippold GM, Frary SG, Abshire DA, Wilson DK. Improving recruitment, retention, and cultural saliency of health promotion efforts targeting african american men: a scoping Review. Ann Behav Med. 2021. 10.1093/abm/kaab079 PMC9242543 34473823

[45] Resnicow K, Baranowski T, Ahluwalia JS, Braithwaite RL. Cultural sensitivity in public health: Defined and demystified. Ethn Dis. 1999. 10355471

[46] Palmer KNB, Rivers PS, Melton FL (2021). Health promotion interventions for African Americans delivered in U.S. barbershops and hair salons- a systematic review. BMC Public Health.

[47] Brownson RC, Kumanyika SK, Kreuter MW, Haire-Joshu D (2021). Implementation science should give higher priority to health equity. Implement Sci.

[48] Scriven M (1994). The fine line between evaluation and explanation. Eval Pract.

[49] Watson AK, Hernandez BF, Kolodny-Goetz J (2022). Using Implementation Mapping to Build Organizational Readiness. Front Public Health.

[50] Kenworthy T, Domlyn A, Scott VC, Schwartz R, Wandersman A (2023). A proactive, systematic approach to building the capacity of technical assistance providers. Health Promot Pract.

[51] Abshire DA, Wippold GM, Wilson DK, Pinto BM, Probst JC, Hardin JW. Rurality, Gender, and Obesity: An Intersectionality Perspective on Rural Men’s Health. Am J Public Health. 2021;111(10):1761–3. 10.2105/AJPH.2021.306482 PMC8561182 34529501

[52] Miller CE, Vasan RS (2021). The southern rural health and mortality penalty: a review of regional health inequities in the United States. Soc Sci Med.

[53] Wippold GM, Frary SG. The Role of Modifiable, Self-Empowerment-Oriented Variables to Promote Health-Related Quality of Life Among Inadequately Insured Americans. J Primary Prev. 2021;43(1):95–110. 10.1007/s10935-021-00652-1 PMC8924987 34773547

[54] Hekler E, Tiro JA, Hunter CM, Nebeker C. Precision Health: The Role of the Social and Behavioral Sciences in Advancing the Vision. Ann Behav Med. 2020;54(11):805–26. 10.1093/abm/kaaa018 PMC7646154 32338719

[55] Wallen GR, Mitchell SA, Melnyk B (2010). Implementing evidence-based practice: effectiveness of a structured multifaceted mentorship programme. J Adv Nurs.

