# Supplementary material for: Assessing organizational readiness for the Clean Cuts and Sharp Minds Collective: a barbershop health promotion network
Source: Implement Sci Commun. 2024 Apr 16;5:42. doi: 10.1186/s43058-024-00584-x (PMC11022399; doi:10.1186/s43058-024-00584-x)
Supplement: Supplementary file 1 — Supplementary Material 1. [file 43058_2024_584_MOESM1_ESM.docx]

Supplementary File 1

| Domain | Statement to Assess Domain |
| --- | --- |
| Motivation | |
| Relative Advantage | Promoting health at our barbershop seems better than focusing solely on the cut or talking about other things. |
| Compatibility | Promoting health at our barbershop fits with both our barbershop’s mission and our clients’ needs. |
| Simplicity | It seems easy to promote health at our barbershop. |
| Ability to Pilot | We can try to promote health at our barbershop on a small scale to see how it goes. |
| Observability | Over time, we will be able to see how promoting health at our barbershop helps our clients be healthier. |
| Priority | Promoting health at our barbershop is a priority. |
| Innovation-specific Capacity | |
| Innovation-specific Knowledge & Skills | Our barbershop has the ability to promote health among our clients. |
| Champion | We have staff at our barbershop that could support us all learning how to promote health among our clients. |
| Supportive Climate | Our barbershop has the time and interest to learn how to promote health among our clients. |
| Inter-organizational Relationships | We have good relationships with other businesses or nearby organizations that could help us to promote health among our clients. |
| Intra-organizational Relationships | Our staff could support each other in promoting health among our clients. |
| General Capacity | |
| Culture | Our barbershop has a shared vision, mission, and way of doing things. |
| Climate | Staff in our barbershop feel good about working here. |
| Innovativeness | Our barbershop is open to improvement. |
| Resource Utilization | Our barbershop has resources (e.g., money, equipment, space) that can be utilized. |
| Leadership | Our barbershop has good leaders. |
| Internal Operations | Our barbershop has good communication and generally functions well. |
| Staff Capacities | Our barbershop has enough of the right kind of staff to get things done. |
